# Dietary spermidine intake is not associated with prostate cancer incidence or prostate cancer-specific mortality: findings from the prostate, lung, colorectal, and ovarian cancer screening trial

**DOI:** 10.3389/fnut.2026.1775582

**Published:** 2026-07-29

**Authors:** Guilin Wang, Jiayi Lin, Zhifeng Lin, Jinhui Jian, Jiongxuan Xu, Jie Xie, Shun Liu, Qingquan Chen, Dewen Zhong

**Affiliations:** 1 Longyan First Affiliated Hospital of Fujian Medical University, Urology and Pediatric Surgery, Longyan, Fujian, China; 2 Fujian Medical University, Fuzhou, Fujian, China; 3 School of Biological Science and Medical Engineering and Beijing Advanced Innovation Centre for Biomedical Engineering, Beihang University, Beijing, China

**Keywords:** cancer mortality, dietary intake, non-linear association, PLCO trial, polyamines, prostate cancer, spermidine

## Abstract

**Background:**

The role of dietary spermidine in prostate cancer outcomes remains unclear. We examined associations between spermidine intake and prostate cancer incidence, all-cause mortality, and prostate cancer-specific mortality in a large prospective cohort.

**Methods:**

This study included 54,517 male participants from the PLCO cancer screening trial. Spermidine in the diet was assessed through a food frequency questionnaire. Incidence was assessed using logistic regression, all-cause mortality using Cox proportional hazards models, and prostate cancer-specific mortality using Fine-Gray competing risk models. Multivariable models adjusted for available demographic, lifestyle, and clinical covariates; total energy intake was not included because it could not be reliably calculated from the processed analytic dataset.

**Results:**

During the follow-up period, a total of 6,643 cases of prostate cancer and 603 cases of prostate cancer-related deaths were observed. The intake of spermidine was not associated with the incidence of prostate cancer (the relative risk value between the highest quintile and the lowest quintile was 1.00, with a 95% confidence interval of 0.89–1.12). A non-linear inverse association was observed in terms of all-cause mortality (non-linear *p* value < 0.010). However, in the competing risk model, no significant association was found with the specific mortality rate of prostate cancer (the relative risk value of the highest quintile was 1.24, with a 95% confidence interval of 0.96–1.60, *p* = 0.10).

**Conclusion:**

Dietary spermidine intake is not associated with prostate cancer incidence or prostate cancer-specific mortality. The observed inverse association with all-cause mortality may reflect residual confounding. These findings are hypothesis-generating and do not support clinical or dietary recommendations.

## 1 Introduction

Prostate cancer, one of the most common malignant tumors in men worldwide, is a major public health challenge (1). Epidemiological data indicate that prostate cancer ranks second in global incidence among male cancers (2, 3), with over 390,000 deaths reported globally in 2022. The disease profoundly impacts men’s life expectancy and quality of life. Despite significant advances in diagnosis (4, 5) and treatment (6), managing advanced prostate cancer, particularly castration-resistant prostate cancer (CRPC), remains challenging (7).

The development and progression of prostate cancer involve a complex multifactorial process, including age, family history, genetic predisposition, environmental exposures, and lifestyle factors (8). In exploring strategies for prostate cancer prevention and treatment, dietary factors (9, 10) have gained increasing attention as modifiable risk elements. Among these, the role of polyamine metabolic pathways in prostate cancer biology is of particular interest (11). Small polyamine molecules, including putrescine, spermidine and spermine, are abundant in prostate tissue (12). Research has indicated that elevated intracellular levels of polyamines are a characteristic manifestation in the prostate; however, dysregulation of polyamine metabolism may lead to rapid cellular proliferation, thereby promoting the progression of prostate cancer (13). This also suggests that spermidine may play a role in prostate cancer, and the study by Tobias Eisenberg et al. also mentioned that spermidine may have the function of inhibiting tumor progression (14).

Spermidine, a naturally occurring polyamine with autophagy-inducing, antioxidant (14), and immune-modulating properties (15), plays a critical role in cellular growth, proliferation, and autophagy processes. Spermidine is a well-established inducer of autophagy, a lysosomal degradation pathway critical for maintaining cellular homeostasis. In prostate cancer, autophagy can have dual roles. Autophagy may play either harmful or protective roles in different cellular contexts. That is, in certain circumstances, autophagy may promote tumor growth and progression (16). However, in specific contexts, spermidine-induced autophagy is associated with tumor-suppressive effects, such as clearing damaged organelles and inhibiting malignant progression (17). Recent groundbreaking studies reveal that spermidine metabolism is intricately linked to the tumor immune microenvironment. By modulating immune cell function and enhancing immunogenic cell death, spermidine can reinvigorate anti-tumor immunity, offering a novel therapeutic angle for prostate cancer (18, 19).

Nevertheless, the specific mechanisms of spermidine in prostate cancer remain to be fully elucidated. Recent studies have highlighted an important link between polyamine metabolism and the tumor immune microenvironment. An innovative study demonstrated that targeting polyamine metabolism can simultaneously induce oxidative/carbonyl stress and inhibit the endogenous antioxidant system. This is achieved by converting the high concentrations of spermidine present in prostatic fluid into hydrogen peroxide and acrolein, thereby synergistically amplifying oxidative/carbonyl damage, triggering potent immunogenic cell death (ICD), and significantly enhancing the recruitment of mature dendritic cells and T cells within the tumor microenvironment. This process ultimately inhibits prostate cancer metastasis and recurrence (16), providing a new perspective for understanding spermidine’s role in this malignancy.

Despite these important scientific insights, large-scale population studies on the relationship between dietary spermidine intake and the risk of prostate cancer incidence and mortality remain limited. In particular, there is a lack of in-depth research on how spermidine intake affects survival in patients already diagnosed with prostate cancer. Based on the Prostate, Lung, Colorectal, and Ovarian (PLCO) Cancer Screening Trial database, this study aims to assess whether dietary spermidine intake is associated with prostate cancer incidence and mortality. Furthermore, it seeks to explore the relationship between dietary spermidine intake and the risk of death among prostate cancer patients, thereby seeking to offer suggestions for dietary interventions and adjuvant therapy strategies for prostate cancer.

## 2 Methods

### 2.1 Study design and population

**Figure 1:**
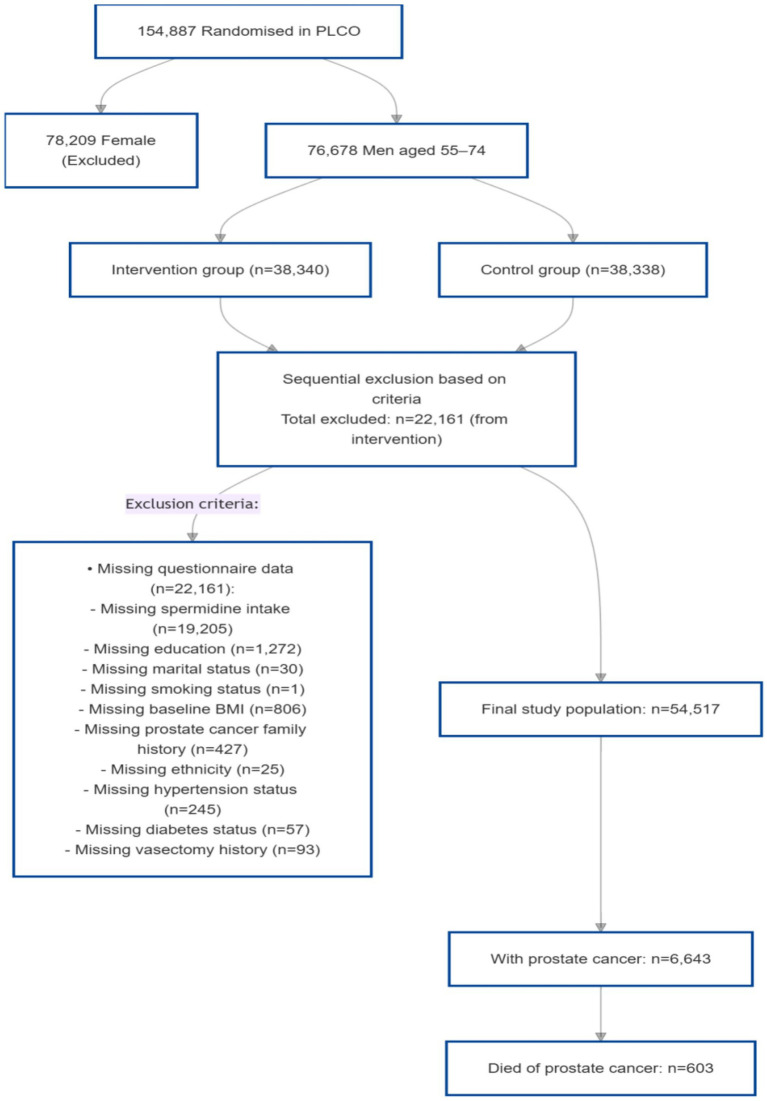
PLCO participant flow chart.

Of the 154,887 participants in the study, 76,678 men aged 55–74 years were randomly assigned to the intervention group (*n* = 38,340) and the control group (*n* = 38,338) in the PLCO trial. In this study, because the outcome was prostate cancer, only men were considered. We excluded 14,935 men from all those assigned to the intervention group according to the following selection criteria: 1. Missing questionnaire information (*n* = 22,161): missing spermidine intake (*n* = 19,205), missing education information (*n* = 1,272), missing marital information (*n* = 30), missing smoking information (*n* = 1), missing baseline BMI (*n* = 806), missing family history of prostate cancer (*n* = 427), missing ethnicity information (*n* = 25), missing hypertension information (*n* = 245), missing diabetes information (*n* = 57), and missing vasectomy information (*n* = 93). A total of 54,517 participants were enrolled, including 6,643 patients with prostate cancer, of whom 603 died from prostate cancer. A detailed participant flow diagram is presented in Figure 1.

The PLCO trial was approved by the Institutional Review Board of the National Cancer Institute (USA), and all participants provided written informed consent (Project ID: PLCO-1221).

### 2.2 Assessment of prostate cancer outcomes

Prostate cancer diagnoses were ascertained through a combination of annual screening—including prostate-specific antigen (PSA) testing and digital rectal examination in the intervention arm—self-reporting via annual follow-up surveys, and review of medical records and death certificates. Advanced prostate cancer was defined as clinical stage III or IV according to the TNM classification, or a Gleason score ≥ 8.

### 2.3 Dietary assessment

**Figure 2:**
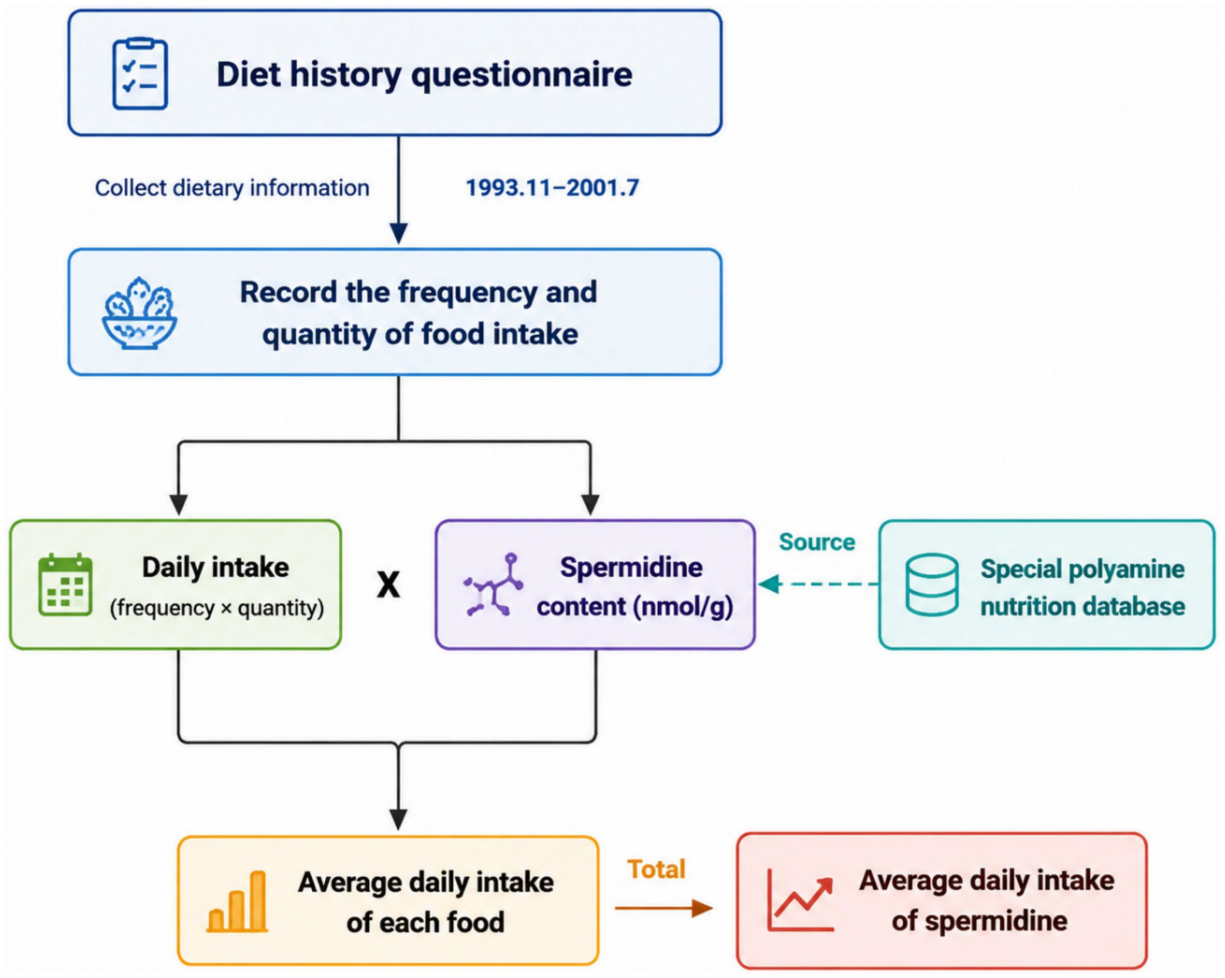
Study flowchart and data processing for spermidine intake assessment.

Dietary intake was assessed using the self-administered Dietary History Questionnaire (DHQ), a validated food-frequency questionnaire developed by the National Cancer Institute. The DHQ includes 124 food items and collects information on portion sizes and dietary supplement use. Total spermidine intake (nmol/day) from dietary sources served as the primary exposure variable. The average daily spermidine intake for each participant, which was used for statistical analysis, was calculated by multiplying the daily intake of each food item by its spermidine content (nmol/g) and then summing the results for all foods (Figure 2). The spermidine content of foods was sourced from a specialized polyamine nutritional database (20, 21).

### 2.4 Statistical analysis

Baseline characteristics of the study population are presented according to quintiles (Q1–Q5) of dietary spermidine intake. Continuous variables are expressed as mean ± standard deviation (SD) and compared using analysis of variance (ANOVA). Categorical variables are presented as frequencies (percentages) and compared using the chi-square test.

Differences in median spermidine intake between prostate cancer cases (total, non-advanced, and advanced) and non-cases were compared using the Wilcoxon rank-sum test.

Incidence was assessed using logistic regression. All-cause mortality was evaluated using Cox proportional hazards regression in the full analytic cohort, with follow-up time accrued from PLCO enrollment to death or censoring. Prostate cancer-specific mortality was evaluated using a Fine-Gray competing risk model among participants diagnosed with prostate cancer, with follow-up time accrued from prostate cancer diagnosis to prostate cancer death or censoring. Spermidine intake was analyzed both as a categorical variable (in quintiles, with Q1 as the reference) and as a continuous variable using restricted cubic splines (RCS) with four knots (placed at the 5th, 35th, 65th, and 95th percentiles) to explore potential non-linear dose–response relationships.

Three multivariable models were constructed:

Model 1 (HR₁): Unadjusted.
Model 2 (HR₂): Adjusted for age, marital status, smoking status, BMI, education, and race.
Model 3 (HR₃): Additionally adjusted for family history of prostate cancer, hypertension, diabetes, and history of vasectomy. Total energy intake was not included as a covariate because it could not be reliably calculated from the processed analytic dataset.

Hazard ratios (HRs) with 95% confidence intervals (CIs) were calculated. A two-sided *p* value < 0.05 was considered statistically significant. All analyses were performed using R software (version 4.2.3).

## 3 Results

### 3.1 Baseline characteristics of the study population

The baseline characteristics of the 54,517 participants, stratified by quintiles of dietary spermidine intake, are presented in Table 1. Among the 54,517 participants in the PLCO trial, most baseline characteristics showed significant differences after grouping by spermidine level quintiles (*p* < 0.05). With increasing spermidine levels (Q1-Q5), BMI gradually decreased, the proportion of never-smokers increased, while the proportion of current smokers decreased, the prevalence of diabetes increased, the proportion of those with postgraduate degrees increased, and the median age at death increased from 79 to 81 years. Simultaneously, there were also inter-group differences in marital status, hypertension, and race (*p* < 0.05). However, no significant differences were observed in randomization, prostate cancer prevalence, or stage among the groups (*p* > 0.05).

**Table 1:** Baseline characteristics of participants stratified by spermidine intake in the PLCO screening trial.

Variables	Total (*n* = 54,517)	Q1 (*n* = 10,904)	Q2 (*n* = 10,903)	Q3 (*n* = 10,903)	Q4 (*n* = 10,903)	Q5 (*n* = 10,904)	*p*
Age, M (Q₁, Q₃)	62.00 (58.00, 67.00)	62.00 (58.00,67.00)	62.00 (58.00,67.00)	62.00 (58.00,67.00)	62.00 (58.00,67.00)	62.00 (58.00,67.00)	0.001
Age group, *n* (%)							0.008
<= 59	17,612 (32.31)	3,683 (33.78)	3,556 (32.61)	3,526 (32.34)	3,409 (31.27)	3,438 (31.53)	
> = 70	6,950 (12.75)	1,369 (12.56)	1,377 (12.63)	1,408 (12.91)	1,424 (13.06)	1,372 (12.58)	
60–64	17,206 (31.56)	3,357 (30.79)	3,386 (31.06)	3,481 (31.93)	3,443 (31.58)	3,539 (32.46)	
65–69	12,749 (23.39)	2,495 (22.88)	2,584 (23.70)	2,488 (22.82)	2,627 (24.09)	2,555 (23.43)	
BMI, M (Q₁, Q₃)	26.96 (24.78, 29.65)	27.12 (25.03,29.79)	27.09 (24.96,29.75)	27.04 (24.89,29.72)	26.96 (24.82,29.56)	26.57 (24.39,29.41)	<0.001
Prostate cancer age, M (Q₁, Q₃)	74.00 (69.00, 78.00)	73.00 (69.00,78.00)	74.00 (69.00,78.00)	74.00 (69.00,78.00)	74.00 (69.00,79.00)	74.00 (69.00,79.00)	<0.001
Mortality age, M (Q₁, Q₃)	80.00 (76.00, 85.00)	79.00 (76.00,84.00)	80.00 (76.00,85.00)	80.00 (76.00,85.00)	81.00 (77.00,85.00)	81.00 (77.00,85.00)	<0.001
Marital status, *n* (%)							<0.001
Divorced	4,170 (7.65)	1,070 (9.81)	804 (7.37)	728 (6.68)	698 (6.40)	870 (7.98)	
Married or living as married	46,385 (85.08)	8,924 (81.84)	9,350 (85.76)	9,455 (86.72)	9,468 (86.84)	9,188 (84.26)	
Never married	1,686 (3.09)	349 (3.20)	309 (2.83)	281 (2.58)	329 (3.02)	418 (3.83)	
Separated	476 (0.87)	113 (1.04)	87 (0.80)	80 (0.73)	99 (0.91)	97 (0.89)	
Widowed	1800 (3.30)	448 (4.11)	353 (3.24)	359 (3.29)	309 (2.83)	331 (3.04)	
Smoking status, *n* (%)							<0.001
Current cigarette smoker	5,674 (10.41)	1,667 (15.29)	1,287 (11.80)	1,054 (9.67)	899 (8.25)	767 (7.03)	
Former cigarette smoker	28,559 (52.39)	5,708 (52.35)	5,723 (52.49)	5,813 (53.32)	5,661 (51.92)	5,654 (51.85)	
Never smoked cigarettes	20,284 (37.21)	3,529 (32.36)	3,893 (35.71)	4,036 (37.02)	4,343 (39.83)	4,483 (41.11)	
Family history of prostate cancer, *n* (%)							<0.001
No	49,617 (91.01)	9,931 (91.08)	9,905 (90.85)	9,924 (91.02)	9,945 (91.21)	9,912 (90.90)	
Possibly	840 (1.54)	207 (1.90)	193 (1.77)	144 (1.32)	137 (1.26)	159 (1.46)	
Yes	4,060 (7.45)	766 (7.02)	805 (7.38)	835 (7.66)	821 (7.53)	833 (7.64)	
Hypertension, *n* (%)							0.016
No	36,372 (66.72)	7,176 (65.81)	7,196 (66.00)	7,303 (66.98)	7,378 (67.67)	7,319 (67.12)	
Yes	18,145 (33.28)	3,728 (34.19)	3,707 (34.00)	3,600 (33.02)	3,525 (32.33)	3,585 (32.88)	
Diabetes, *n* (%)							<0.001
No	50,068 (91.84)	10,103 (92.65)	10,086 (92.51)	10,023 (91.93)	9,972 (91.46)	9,884 (90.65)	
Yes	4,449 (8.16)	801 (7.35)	817 (7.49)	880 (8.07)	931 (8.54)	1,020 (9.35)	
Vasectomy, *n* (%)							<0.001
No	39,043 (71.62)	7,751 (71.08)	7,621 (69.90)	7,746 (71.04)	7,840 (71.91)	8,085 (74.15)	
Yes	15,474 (28.38)	3,153 (28.92)	3,282 (30.10)	3,157 (28.96)	3,063 (28.09)	2,819 (25.85)	
Randomization arm, n (%)							0.119
Control	25,823 (47.37)	5,276 (48.39)	5,186 (47.56)	5,134 (47.09)	5,091 (46.69)	5,136 (47.10)	
Intervention	28,694 (52.63)	5,628 (51.61)	5,717 (52.44)	5,769 (52.91)	5,812 (53.31)	5,768 (52.90)	
Prostate cancer, *n* (%)							0.112
No	47,874 (87.81)	9,653 (88.53)	9,580 (87.87)	9,557 (87.65)	9,552 (87.61)	9,532 (87.42)	
Yes	6,643 (12.19)	1,251 (11.47)	1,323 (12.13)	1,346 (12.35)	1,351 (12.39)	1,372 (12.58)	
Education, *n* (%)							<0.001
12 Years or completed high school	10,195 (18.70)	2,314 (21.22)	2069 (18.98)	2036 (18.67)	1935 (17.75)	1841 (16.88)	
8–11 years	3,417 (6.27)	821 (7.53)	683 (6.26)	641 (5.88)	611 (5.60)	661 (6.06)	
College graduate	10,555 (19.36)	2015 (18.48)	2,159 (19.80)	2,112 (19.37)	2,156 (19.77)	2,113 (19.38)	
Less than 8 years	522 (0.96)	113 (1.04)	105 (0.96)	87 (0.80)	93 (0.85)	124 (1.14)	
Post high school training other than college	6,885 (12.63)	1,442 (13.22)	1,433 (13.14)	1,392 (12.77)	1,349 (12.37)	1,269 (11.64)	
Postgraduate	12,031 (22.07)	1897 (17.40)	2,177 (19.97)	2,464 (22.60)	2,632 (24.14)	2,861 (26.24)	
Some college	10,912 (20.02)	2,302 (21.11)	2,277 (20.88)	2,171 (19.91)	2,127 (19.51)	2035 (18.66)	
Race, *n* (%)							<0.001
American Indian	112 (0.21)	30 (0.28)	18 (0.17)	14 (0.13)	25 (0.23)	25 (0.23)	
Asian	2,137 (3.92)	683 (6.26)	387 (3.55)	354 (3.25)	323 (2.96)	390 (3.58)	
Black, Non-Hispanic	1,617 (2.97)	389 (3.57)	258 (2.37)	263 (2.41)	282 (2.59)	425 (3.90)	
Hispanic	975 (1.79)	246 (2.26)	185 (1.70)	190 (1.74)	169 (1.55)	185 (1.70)	
Pacific Islander	300 (0.55)	99 (0.91)	52 (0.48)	41 (0.38)	50 (0.46)	58 (0.53)	
White, Non-Hispanic	49,376 (90.57)	9,457 (86.73)	10,003 (91.75)	10,041 (92.09)	10,054 (92.21)	9,821 (90.07)	
Prostate stage, *n* (%)							0.069
None	47,879 (87.82)	9,656 (88.55)	9,581 (87.87)	9,557 (87.65)	9,552 (87.61)	9,533 (87.43)	
Stage I	2,267 (4.16)	432 (3.96)	432 (3.96)	474 (4.35)	456 (4.18)	473 (4.34)	
Stage II	11 (0.02)	1 (0.01)	3 (0.03)	1 (0.01)	4 (0.04)	2 (0.02)	
Stage IIA	2,666 (4.89)	501 (4.59)	537 (4.93)	550 (5.04)	564 (5.17)	514 (4.71)	
Stage IIB	912 (1.67)	165 (1.51)	195 (1.79)	193 (1.77)	167 (1.53)	192 (1.76)	
Stage III	581 (1.07)	106 (0.97)	113 (1.04)	105 (0.96)	117 (1.07)	140 (1.28)	
Stage IV	201 (0.37)	43 (0.39)	42 (0.39)	23 (0.21)	43 (0.39)	50 (0.46)	

### 3.2 Association between prostate cancer incidence and spermidine

According to the logistic regression analysis results in Table 2, in the coarse model (Model 1) without adjusting for confounding factors, compared with the lowest quintile of spermidine intake (Q1), the risk of prostate cancer was significantly increased in the higher intake groups (Q3, Q4, Q5) (Q3: OR = 1.09, 95%CI 1.01 ~ 1.18, *p* = 0.047; Q4: OR = 1.09, 95%CI 1.01 ~ 1.18, *p* = 0.037; Q5: OR = 1.11, 95%CI 1.02 ~ 1.21, *p* = 0.012). However, after adjusting for age, marital status, smoking status, BMI, education level, and race (Model 2), and further adjusting for factors such as family history of prostate cancer, hypertension, diabetes, and vasectomy (Model 3), the odds ratios (ORs) for all spermidine intake quintiles (Q2–Q5) compared to Q1 were close to 1.0, and the *p*-values were all greater than 0.05, indicating no statistical significance. These results suggest that, after controlling for potential confounding factors, no independent association was found between dietary spermidine intake and the risk of prostate cancer in the general population.

**Table 2:** Logistic regression analysis of prostate cancer and spermidine intake in the general population.

Variables	Model 1		Model 2		Model 3	
	OR (95% CI)	*p*	OR (95% CI)	*p*	OR (95% CI)	*p*
Spermidine five						
Q1	1.00 (Reference)		1.00 (Reference)		1.00 (Reference)	
Q2	1.07 (0.98 ~ 1.16)	0.13	1.03 (0.95 ~ 1.12)	0.513	1.03 (0.95 ~ 1.12)	0.513
Q3	1.09 (1.01 ~ 1.18)	0.047	1.04 (0.95 ~ 1.12)	0.412	1.04 (0.95 ~ 1.13)	0.395
Q4	1.09 (1.01 ~ 1.18)	0.037	1.02 (0.94 ~ 1.11)	0.596	1.03 (0.95 ~ 1.12)	0.526
Q5	1.11 (1.02 ~ 1.21)	0.012	1.04 (0.95 ~ 1.13)	0.396	1.04 (0.96 ~ 1.13)	0.312

OR, odds ratio; CI, confidence interval. Model 1: Crude. Model 2: Adjust: age, marital_status, smoking_status, BMI, education, race. Model 3: Adjust: age, marital_status, smoking_status, BMI, family_history_of_prostate_cancer, hypertension, diabetes, vasectomy, education, race.

### 3.3 Association between prostate cancer stage and spermidine

Based on the logistic regression analysis results in Table 3, the association between dietary spermidine intake and prostate cancer stage was analyzed in prostate cancer patients. In the coarse model (Model 1) without adjusting for confounding factors, the risk of higher stage was significantly reduced in group Q3 compared with the lowest quintile of spermidine intake (Q1) (OR = 0.78, 95%CI 0.60–0.99, *p* = 0.046); while there were no statistically significant differences between groups Q2, Q4, and Q5 and group Q1 (*p* > 0.05 for all groups). After adjusting for age, marital status, smoking status, BMI, education level, and race (Model 2), and further adjusting for factors such as family history of prostate cancer, hypertension, diabetes, and vasectomy (Model 3), the results remained consistent: the Q3 group still showed a statistically significant lower risk (Model 2: OR = 0.77, 95% CI 0.60–0.99, *p* = 0.042; Model 3: OR = 0.77, 95% CI 0.60–0.99, *p* = 0.039), while other spermidine intake groups showed no significant association with the Q1 group. These results suggest that in prostate cancer patients, moderate levels (Q3) of spermidine intake may be associated with lower prostate cancer stage (i.e., earlier or lower malignancy), but this association is not linear; further increases in intake (Q4, Q5) did not show lower OR values.

**Table 3:** Logistic regression analysis of prostate cancer stage and spermidine intake in patients with prostate cancer.

Variables	Model 1		Model 2		Model 3	
	OR (95% CI)	*p*	OR (95% CI)	*p*	OR (95% CI)	*p*
Spermidine five						
Q1	1.00 (Reference)		1.00 (Reference)		1.00 (Reference)	
Q2	0.98 (0.77 ~ 1.24)	0.866	0.98 (0.77 ~ 1.25)	0.873	0.98 (0.77 ~ 1.24)	0.849
Q3	0.78 (0.60 ~ 0.99)	0.046	0.77 (0.60 ~ 0.99)	0.042	0.77 (0.60 ~ 0.99)	0.039
Q4	0.99 (0.78 ~ 1.26)	0.94	0.99 (0.78 ~ 1.26)	0.952	0.99 (0.78 ~ 1.25)	0.904
Q5	1.19 (0.94 ~ 1.49)	0.144	1.20 (0.95 ~ 1.51)	0.128	1.20 (0.95 ~ 1.51)	0.129

OR, odds ratio; CI, confidence interval. Model 1: Crude. Model 2: Adjust: age, marital_status, smoking_status, BMI, education, race. Model 3: Adjust: age, marital_status, smoking_status, BMI, family_history_of_prostate_cancer, hypertension, diabetes, vasectomy, education, race.

### 3.4 Association between all-cause mortality and spermidine

Based on the Cox proportional hazards regression analysis results in Table 4, the association between dietary spermidine intake and all-cause mortality risk was assessed in the full analytic cohort from PLCO enrollment, with a median follow-up of 14.41 years and total person-years of 1,778,221. In the coarse model (Model 1) without adjusting for confounding factors, the all-cause mortality risk was significantly lower in groups Q2–Q5 compared to the lowest quintile of spermidine intake (Q1) (Q2: HR = 0.91, 95%CI 0.88–0.95, *p* < 0.001; Q3: HR = 0.87, 0.84–0.91, p < 0.001; Q4: HR = 0.86, 0.83–0.90, p < 0.001; Q5: HR = 0.86, 0.83–0.90, p < 0.001). After adjusting for age, marital status, smoking status, BMI, education level, and race (Model 2), the risk of death (HR) decreased but remained statistically significant (HR range 0.90–0.93 for Q2–Q5, all *p* < 0.001). Further adjustments for family history of prostate cancer, hypertension, diabetes, and vasectomy (Model 3) resulted in stable outcomes (Q2: HR = 0.92, 0.88–0.96; Q3: HR = 0.89, 0.86–0.93; Q4: HR = 0.89, 0.85–0.93; Q5: HR = 0.89, 0.86–0.93; all p < 0.001). These results indicate that higher dietary spermidine intake is significantly associated with a reduced risk of all-cause mortality, and this association remains robust after adjusting for various confounding factors.

**Table 4:** Cox proportional hazards regression model of all-cause mortality and spermidine intake.

Variables	Cases	Person-year	Model 1		Model 2		Model 3	
			HR (95%CI)	*p*	HR (95%CI)	*p*	HR (95%CI)	*p*
Spermidine five								
Q1	4,767	375,339	1.00 (Reference)		1.00 (Reference)		1.00 (Reference)	
Q2	4,511	357,827	0.91 (0.88 ~ 0.95)	<0.001	0.93 (0.89 ~ 0.96)	<0.001	0.92 (0.88 ~ 0.96)	<0.001
Q3	4,372	348,152	0.87 (0.84 ~ 0.91)	<0.001	0.90 (0.87 ~ 0.94)	<0.001	0.89 (0.86 ~ 0.93)	<0.001
Q4	4,325	345,432	0.86 (0.83 ~ 0.90)	<0.001	0.90 (0.86 ~ 0.94)	<0.001	0.89 (0.85 ~ 0.93)	<0.001
Q5	4,367	351,471	0.86 (0.83 ~ 0.90)	<0.001	0.91 (0.88 ~ 0.95)	<0.001	0.89 (0.86 ~ 0.93)	<0.001

HR: hazard ratio, CI: confidence interval. Model 1: Crude. Model 2: Adjust: age, marital_status, smoking_status, BMI, education, race. Model 3: Adjust: age, marital_status, smoking_status, bmi, family_history_of_prostate_cancer, hypertension, diabetes, vasectomy, education, race.

The proportional risk hypothesis test (based on Schoenfeld residuals) of the Cox model showed that the primary study variable, spermidine intake quintiles, met the proportional risk hypothesis (χ^2^ = 7.6801, df = 4, *p* = 0.1040). Some covariates, such as age (*p* < 0.001), marital status (*p* = 0.0021), and smoking status (p < 0.001), had *p*-values <0.05, suggesting a possible time effect. However, although the global test (GLOBAL) was significant (χ^2^ = 105.85, df = 32, *p* < 0.001), given that the primary exposure variable met the hypothesis, the estimated hazard ratio (HR) based on this variable remained stable over time, indicating a reliable conclusion.

### 3.5 Regression analysis of competition risk between prostate cancer mortality and spermidine

Based on the Fine-Gray competing hazard regression model analysis results in Table 5, the association between dietary spermidine intake and prostate cancer-specific mortality risk was assessed among participants diagnosed with prostate cancer, with follow-up counted from prostate cancer diagnosis (median follow-up 15.14 years; total person-years 48,424), while considering competing events from other causes. The results showed that in all three models, the estimated hazard ratios (HRs) for groups Q2–Q5 were not statistically significant compared to the lowest quintile of spermidine intake (Q1).

**Table 5:** Fine-Gray competitive risk regression analysis of prostate cancer mortality and spermidine intake.

Variables	Cases	Person-year	Model 1		Model 2		Model 3	
			HR (95%CI)	*p*	HR (95%CI)	*p*	HR (95%CI)	*p*
Spermidine five								
Q1	109	8,847	1.00 (Reference)		1.00 (Reference)		1.00 (Reference)	
Q2	117	9,474	1.06 (0.82–1.37)	0.67	1.04 (0.80–1.35)	0.76	1.04 (0.80–1.35)	0.76
Q3	111	8,879	0.99 (0.77–1.30)	1	0.97 (0.74–1.27)	0.84	0.97 (0.75–1.27)	0.85
Q4	123	9,845	1.11 (0.86–1.44)	0.42	1.07 (0.82–1.39)	0.61	1.07 (0.83–1.40)	0.59
Q5	143	11,379	1.27 (0.99–1.63)	0.06	1.22 (0.95–1.58)	0.12	1.24 (0.96–1.60)	0.10

HR, hazard ratio; CI, confidence interval. Model 1: Crude. Model 2: Adjust: age, marital_status, smoking_status, BMI, education, race. Model 3: Adjust: age, marital_status, smoking_status, BMI, family_history_of_prostate_cancer, hypertension, diabetes, vasectomy, education, race.

In Model 1, which was not adjusted for confounding factors, the HR for group Q5 was 1.27 (95% CI 0.99–1.63, *p* = 0.06), with no significant marginal value; the HRs for groups Q2, Q3, and Q4 were 1.06, 0.99, and 1.11, respectively, with all *p* values> 0.05. After adjusting for age, marital status, smoking status, BMI, education level, and race (Model 2), the HR for Q5 was 1.22 (0.95–1.58, *p* = 0.12). After further adjusting for factors such as family history of prostate cancer, hypertension, diabetes, and vasectomy (Model 3), the HR for Q5 was 1.24 (0.96–1.60, *p* = 0.10), which remained statistically insignificant. The adjusted HRs for all other groups were close to 1.0, with *p* values > 0.05.

These results indicate that there was no independent statistical association between dietary spermidine intake and the risk of death from prostate cancer in this study population. Although the Q5 group showed a certain trend of increased risk, it did not reach the significance threshold (*p* < 0.05) (Table 6).

**Table 6:** Subgroup analysis of prostate cancer staging and spermidine intake in patients with prostate cancer.

Variables	*n* (%)	Q1	Q2	OR (95% CI)	*p*	P for interaction
All patients	54,517 (100.00)	2574/21807	4069/32710	1.06 (1.01 ~ 1.12)	0.026	
Age group						0.663
<= 59	17,612 (32.31)	630/7239	915/10373	1.01 (0.91 ~ 1.13)	0.785	
60–64	17,206 (31.56)	876/6743	1441/10463	1.07 (0.98 ~ 1.17)	0.143	
65–69	12,749 (23.39)	703/5079	1150/7670	1.10 (0.99 ~ 1.22)	0.071	
> = 70	6,950 (12.75)	365/2746	563/4204	1.01 (0.88 ~ 1.16)	0.905	
Marital status						0.894
Divorced	4,170 (7.65)	171/1874	230/2296	1.11 (0.90 ~ 1.37)	0.331	
Married or living as married	46,385 (85.08)	2234/18274	3592/28111	1.05 (0.99 ~ 1.11)	0.079	
Never married	1,686 (3.09)	60/658	95/1028	1.01 (0.72 ~ 1.42)	0.932	
Separated	476 (0.87)	19/200	35/276	1.38 (0.77 ~ 2.50)	0.282	
Widowed	1800 (3.30)	90/801	117/999	1.05 (0.78 ~ 1.40)	0.753	
Smoking status						0.731
Current cigarette smoker	5,674 (10.41)	276/2954	280/2720	1.11 (0.93 ~ 1.33)	0.229	
Former cigarette smoker	28,559 (52.39)	1312/11431	2020/17128	1.03 (0.96 ~ 1.11)	0.415	
Never smoked cigarettes	20,284 (37.21)	986/7422	1769/12862	1.04 (0.96 ~ 1.13)	0.348	
BMI group						0.248
0–18.5	155 (0.28)	4/59	11/96	1.78 (0.54 ~ 5.87)	0.344	
18.5–25	14,298 (26.23)	670/5396	1219/8902	1.12 (1.01 ~ 1.24)	0.029	
25–30	27,810 (51.01)	1348/11278	2079/16532	1.06 (0.98 ~ 1.14)	0.121	
30+	12,254 (22.48)	552/5074	760/7180	0.97 (0.86 ~ 1.09)	0.604	
Family history of prostate cancer						0.968
No	49,617 (91.01)	2257/19836	3557/29781	1.06 (1.00 ~ 1.12)	0.055	
Possibly	840 (1.54)	44/400	51/440	1.06 (0.69 ~ 1.63)	0.787	
Yes	4,060 (7.45)	273/1571	461/2489	1.08 (0.92 ~ 1.27)	0.356	
Hypertension						0.617
No	36,372 (66.72)	1726/14372	2760/22000	1.05 (0.99 ~ 1.12)	0.129	
Yes	18,145 (33.28)	848/7435	1309/10710	1.08 (0.99 ~ 1.19)	0.095	
Diabetes						0.684
No	50,068 (91.84)	2436/20189	3823/29879	1.07 (1.01 ~ 1.13)	0.016	
Yes	4,449 (8.16)	138/1618	246/2831	1.02 (0.82 ~ 1.27)	0.855	
Vasectomy						0.198
No	39,043 (71.62)	1848/15372	2941/23671	1.04 (0.98 ~ 1.10)	0.236	
Yes	15,474 (28.38)	726/6435	1128/9039	1.12 (1.02 ~ 1.24)	0.024	
Randomization arm						0.429
Control	25,823 (47.37)	1215/10462	1842/15361	1.04 (0.96 ~ 1.12)	0.356	
Intervention	28,694 (52.63)	1359/11345	2227/17349	1.08 (1.01 ~ 1.16)	0.032	
Education						0.861
12 years or completed high school	10,195 (18.70)	543/4383	725/5812	1.01 (0.89 ~ 1.14)	0.897	
8–11 Years	3,417 (6.27)	160/1504	225/1913	1.12 (0.90 ~ 1.39)	0.303	
College graduate	10,555 (19.36)	508/4174	795/6381	1.03 (0.91 ~ 1.16)	0.66	
Less than 8 years	522 (0.96)	20/218	38/304	1.41 (0.80 ~ 2.51)	0.235	
Post high school training other than college	6,885 (12.63)	311/2875	476/4010	1.11 (0.95 ~ 1.29)	0.176	
Postgraduate	12,031 (22.07)	509/4074	1052/7957	1.07 (0.95 ~ 1.20)	0.261	
Some college	10,912 (20.02)	523/4579	758/6333	1.05 (0.94 ~ 1.19)	0.381	
Race						0.129
American Indian	112 (0.21)	3/48	6/64	1.55 (0.37 ~ 6.55)	0.55	
Asian	2,137 (3.92)	94/1070	82/1067	0.86 (0.63 ~ 1.18)	0.355	
Black, Non-Hispanic	1,617 (2.97)	100/647	166/970	1.13 (0.86 ~ 1.48)	0.379	
Hispanic	975 (1.79)	38/431	61/544	1.31 (0.85 ~ 2.00)	0.22	
Pacific Islander	300 (0.55)	8/151	20/149	2.77 (1.18 ~ 6.51)	0.019	
White, Non-Hispanic	49,376 (90.57)	2331/19460	3734/29916	1.05 (0.99 ~ 1.11)	0.096	

OR, odds ratio; CI, confidence interval.

### 3.6 Subgroup analysis of prostate cancer stage and spermidine intake

The subgroup analysis results showed that in all 54,517 prostate cancer patients, higher levels of spermidine intake were significantly associated with an increased risk of prostate cancer stage (OR = 1.06, 95% CI: 1.01–1.12, *p* = 0.026). After stratification by age, marital status, smoking status, hypertension, diabetes, randomization group, education level, and race, there were no statistically significant interactions among the subgroups (all interaction *p* values > 0.05). However, significant associations were observed in some subgroups: in patients with a BMI of 18.5–25 kg/m^2^, spermidine intake was positively correlated with the risk of stage (OR = 1.12, 95% CI: 1.01–1.24, *p* = 0.029); the association was also significant in those with a history of vasectomy (OR = 1.12, 95% CI: 1.02–1.24, *p* = 0.024); and significant positive correlations were also observed in the intervention group (OR = 1.08, 95% CI: 1.01–1.16, *p* = 0.032) and among Pacific Islander ethnicity (OR = 2.77, 95% CI: 1.18–6.51, *p* = 0.019). No significant associations were observed in other subgroups such as those aged ≥ 70 years, BMI ≥ 30 kg/m^2^, those with diabetes or hypertension, and the effects of each stratification factor did not show obvious modification effects.

### 3.7 Subgroup analysis of all-cause mortality and spermidine intake

The subgroup analysis results for all-cause mortality (Table 7) showed that in all 54,517 patients, higher spermidine intake was associated with a significantly reduced risk of all-cause mortality (OR = 0.90, 95% CI: 0.88–0.93, *p* < 0.001). When stratified by age, a significant protective association was observed in all age groups (≤ 59 years: OR = 0.84; 60–64 years: OR = 0.93; 65–69 years: OR = 0.87; ≥ 70 years: OR = 0.89; all *p* < 0.05), with an interaction *p* = 0.068, suggesting that age did not significantly modify the effect. In terms of marital status, married/cohabiting and never-married individuals had significantly lower mortality risks (p < 0.05), while those in the divorced, separated or widowed groups had no statistically significant association (interaction *p* = 0.716). Regarding smoking history, only former smokers showed a significant protective effect (OR = 0.91), while current smokers and never-smokers did not reach statistical significance (interaction *p* = 0.069). There was a significant interaction between BMI groups (*p* = 0.003), except for the extremely low BMI group (0–18.5), and all other BMI subgroups (18.5–25, 25–30, ≥ 30) showed significant protective associations, with the normal BMI subgroup having the strongest effect (OR = 0.84). The protective effect of spermidine intake was generally consistent across various subgroups (most subgroups had OR < 1 and *p* < 0.05), and the interaction tests were not significant (*p* > 0.05). In terms of racial subgroups, only in whites was a significant protective association observed (OR = 0.89), while the OR estimates for other races (American Indians, Asians, Blacks, Hispanics, Pacific Islanders) were not statistically significant (interaction *p* = 0.287). Overall, the protective effect of spermidine intake on all-cause mortality was robust in most subgroups, but did not reach statistical significance in specific populations (such as current smokers, extremely low-weight individuals, certain racial groups).

**Table 7:** Subgroup analysis of all-cause mortality and spermidine intake.

Variables	*n* (%)	Q1	Q2	OR (95% CI)	*p*	P for interaction
All patients	54,517 (100.00)	9278/21807	13,094/32710	0.90 (0.88 ~ 0.93)	<0.001	
Age group						0.068
<= 59	17,612 (32.31)	1637/7239	2022/10373	0.84 (0.79 ~ 0.90)	<0.001	
60–64	6,950 (12.75)	2081/2746	3152/4204	0.93 (0.88 ~ 0.99)	0.017	
65–69	17,206 (31.56)	2620/6743	3701/10463	0.87 (0.83 ~ 0.92)	<0.001	
> = 70	12,749 (23.39)	2940/5079	4219/7670	0.89 (0.85 ~ 0.94)	<0.001	
Marital status						0.716
Divorced	4,170 (7.65)	852/1874	994/2296	0.92 (0.84 ~ 1.01)	0.074	
Married or living as married	46,385 (85.08)	7498/18274	10,909/28111	0.91 (0.88 ~ 0.94)	<0.001	
Never married	1,686 (3.09)	335/658	454/1028	0.83 (0.72 ~ 0.96)	0.011	
Separated	476 (0.87)	98/200	136/276	0.92 (0.71 ~ 1.20)	0.554	
Widowed	1800 (3.30)	495/801	601/999	0.94 (0.84 ~ 1.06)	0.33	
Smoking status						0.069
Current cigarette smoker	5,674 (10.41)	1652/2954	1507/2720	0.96 (0.90 ~ 1.03)	0.263	
Former cigarette smoker	28,559 (52.39)	5070/11431	7165/17128	0.91 (0.88 ~ 0.95)	<0.001	
Never smoked cigarettes	20,284 (37.21)	2556/7422	4422/12862	0.97 (0.93 ~ 1.02)	0.296	
BMI group						0.003
0–18.5	155 (0.28)	35/59	47/96	0.80 (0.52 ~ 1.24)	0.324	
18.5–25	14,298 (26.23)	2443/5396	3602/8902	0.84 (0.80 ~ 0.88)	<0.001	
25–30	27,810 (51.01)	4478/11278	6374/16532	0.94 (0.91 ~ 0.98)	0.002	
30+	12,254 (22.48)	2322/5074	3071/7180	0.91 (0.86 ~ 0.96)	<0.001	
Family history of prostate cancer						0.675
No	49,617 (91.01)	8435/19836	11,916/29781	0.90 (0.88 ~ 0.93)	<0.001	
Possibly	840 (1.54)	198/400	200/440	0.89 (0.73 ~ 1.08)	0.241	
Yes	4,060 (7.45)	645/1571	978/2489	0.95 (0.86 ~ 1.04)	0.269	
Hypertension						0.75
No	36,372 (66.72)	5491/14372	7884/22000	0.90 (0.87 ~ 0.94)	<0.001	
Yes	18,145 (33.28)	3787/7435	5210/10710	0.91 (0.88 ~ 0.95)	<0.001	
Diabetes						0.203
No	50,068 (91.84)	8250/20189	11,348/29879	0.89 (0.87 ~ 0.92)	<0.001	
Yes	4,449 (8.16)	1028/1618	1746/2831	0.94 (0.87 ~ 1.02)	0.119	
Vasectomy						0.665
No	39,043 (71.62)	6976/15372	10,088/23671	0.90 (0.88 ~ 0.93)	<0.001	
Yes	15,474 (28.38)	2302/6435	3006/9039	0.89 (0.84 ~ 0.94)	<0.001	
Randomization arm						0.951
Control	25,823 (47.37)	4542/10462	6272/15361	0.91 (0.87 ~ 0.94)	<0.001	
Intervention	28,694 (52.63)	4736/11345	6822/17349	0.90 (0.87 ~ 0.94)	<0.001	
Education						0.734
12 years or completed high school	10,195 (18.70)	1947/4383	2450/5812	0.93 (0.88 ~ 0.99)	0.023	
8–11 years	3,417 (6.27)	807/1504	969/1913	0.91 (0.83 ~ 1.00)	0.045	
College graduate	10,555 (19.36)	1694/4174	2387/6381	0.88 (0.83 ~ 0.94)	<0.001	
Less than 8 years	522 (0.96)	127/218	189/304	1.02 (0.81 ~ 1.28)	0.874	
Post high school training other than college	6,885 (12.63)	1256/2875	1659/4010	0.91 (0.85 ~ 0.98)	0.014	
Postgraduate	12,031 (22.07)	1469/4074	2781/7957	0.93 (0.87 ~ 0.99)	0.025	
Some college	10,912 (20.02)	1978/4579	2659/6333	0.95 (0.90 ~ 1.01)	0.077	
Race						0.287
American Indian	112 (0.21)	26/48	26/64	0.70 (0.41 ~ 1.21)	0.207	
Asian	2,137 (3.92)	391/1070	386/1067	1.01 (0.88 ~ 1.16)	0.887	
Black, Non-Hispanic	1,617 (2.97)	317/647	473/970	0.96 (0.83 ~ 1.11)	0.588	
Hispanic	975 (1.79)	138/431	180/544	1.02 (0.82 ~ 1.27)	0.866	
Pacific Islander	300 (0.55)	67/151	68/149	1.00 (0.71 ~ 1.40)	0.993	
White, Non-Hispanic	49,376 (90.57)	8339/19460	11,961/29916	0.89 (0.87 ~ 0.92)	<0.001	

HR, hazard ratio; CI, confidence interval.

### 3.8 RCS analysis

**Figure 3:**
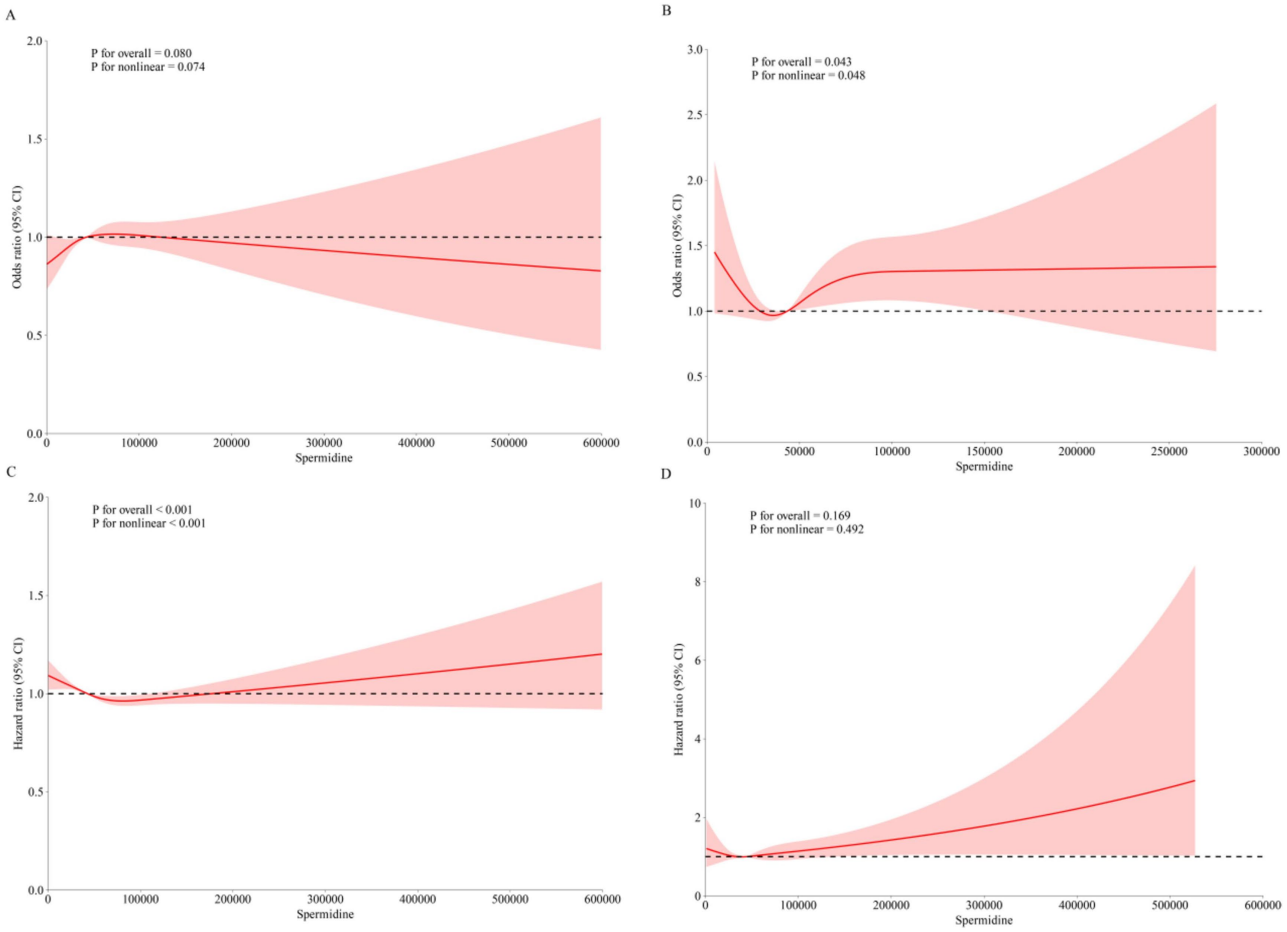
**(A)** RCS curve of prostate cancer incidence and spermidine; **(B)** RCS curve of prostate cancer stage and spermidine; **(C)** RCS curve of all-cause mortality and spermidine; **(D)** RCS curve of prostate cancer mortality and spermidine.

Restricted cubic spline (RCS) analysis was used to analyze the dose–response relationship between spermidine levels and various outcomes. Four nodes were set for each outcome, located at the 5th, 35th, 65th, and 95th percentiles, with the reference value of the one-way RCS curve used as a reference (Figure 3). Specific fitting information and curve characteristics are as follows: A. RCS curve of prostate cancer incidence and spermidine: node values 14159.72, 33910.06, 53159.60, 105790.24, reference value 42721.68. B. RCS curve of prostate cancer stage and spermidine: node values 14348.80, 34524.86, 53998.93, 105579.23, reference value 43351.45. C. RCS curves of all-cause mortality and spermidine: node values were 14159.72, 33910.06, 53159.60, and 105790.24, with a reference value of 42721.68. D. RCS curves of prostate cancer mortality and spermidine: node values were 14627.45, 34587.90, 53623.44, and 106456.96, with a reference value of 43270.64. Among these, spermidine intake, after determining prostate cancer stage, showed a non-linear relationship with all-cause mortality; the other relationships were not statistically significant.

## 4 Discussion

Based on the large-scale prospective cohort platform of the Prostate, Lung, Colorectal, and Ovarian (PLCO) Cancer Screening Trial, this study systematically evaluated the relationship between dietary spermidine intake and prostate cancer incidence, disease stage, all-cause mortality, and prostate cancer-specific mortality. We observed that dietary spermidine intake was not significantly associated with the risk of prostate cancer incidence after adjustment for available demographic, lifestyle, and clinical confounders. In contrast, a non-linear inverse association was observed with prostate cancer stage: participants with moderate spermidine intake (the third quintile) had a significantly lower risk of presenting with higher-stage disease (i.e., less advanced tumors) compared with the lowest intake group. This association was not linear, as no further benefit was seen at higher intake levels (Q4, Q5). Additionally, higher spermidine intake was consistently associated with a reduced risk of all-cause mortality, a finding that persisted across multiple adjustment models. Important caution is warranted: we found no independent association between dietary spermidine intake and prostate cancer-specific mortality in Fine-Gray competing risk models, although a non-significant trend toward increased risk was noted in the highest quintile. The dissociation between all-cause mortality and prostate cancer-specific mortality, together with the stage-related finding, may reflect residual confounding or selection bias rather than a true biological difference; the underlying mechanisms remain unclear. Despite multivariable adjustment for established confounders, residual confounding remains a major concern. Spermidine-rich foods (e.g., whole grains, legumes, nuts, soy products) are integral components of healthy dietary patterns that are also associated with higher physical activity, non-smoking, and higher socioeconomic status. While we adjusted for smoking, BMI, education, and other factors, unmeasured or imperfectly measured confounders—such as overall dietary quality (e.g., HEI or DASH scores), physical activity, or supplement use—could partly explain the observed inverse associations with all-cause mortality. Thus, the mortality findings should not be interpreted as causal effects of spermidine per se.

Regarding dietary spermidine intake and population health outcomes, several observational studies have reported inverse associations with all-cause or cardiovascular mortality (22–24); For example, a UK Biobank study noted a non-linear relationship between dietary polyamine intake and mortality, introducing the concept of a potential “optimal intake range” (22). These findings are consistent with our observation of a robust, non-linear inverse association between spermidine intake and all-cause mortality. However, these studies examined different endpoints and are therefore not directly comparable to our prostate cancer-specific mortality analysis. Discrepancies also exist; the Japanese Takayama cohort found no significant association between spermidine intake and overall mortality, and even a suggestive positive correlation with cancer mortality in women (25). These inconsistencies may arise from differences in dietary patterns, spermidine food sources, assessment tools, or residual confounding across populations. Our study extends this literature by focusing specifically on prostate cancer outcomes and by simultaneously analyzing incidence, stage, and competing mortality endpoints. The non-monotonic pattern observed for prostate cancer stage—where only the third quintile of spermidine intake was associated with lower stage, with no further benefit at higher intakes—should be interpreted with caution. This pattern could be due to chance, model instability, or the play of random variation given multiple comparisons. Indeed, the overall P for trend across quintiles was not significant (P for trend = 0.32, data not shown), and the isolated significant finding in Q3 may represent a false positive. We therefore emphasize that this stage-related finding is exploratory and requires independent replication.

The non-linear dose–response relationship between spermidine intake and all-cause mortality (P for non-linearity = 0.010), with a plateaued effect beyond the second quintile, suggests that any potential survival benefit may be achieved at moderate intake levels. However, this pattern could also reflect residual confounding or measurement error rather than a true biological threshold.

A growing body of experimental research has explored the biological roles of spermidine, particularly its ability to induce autophagy, modulate immune function, and influence cell death pathways such as ferroptosis (13, 15, 16, 26, 27). However, it is essential to emphasize that these mechanistic insights derive from preclinical models (often using doses far exceeding human dietary intake) and do not directly establish that dietary spermidine intake modulates these pathways in humans. Moreover, polyamine metabolism has dual roles in cancer: enzymes such as ornithine decarboxylase (ODC) are upregulated in many cancers and promote tumorigenesis, suggesting that polyamines can also support malignant growth (28). The observed non-linear pattern in our study where only moderate intake was associated with lower stage, might reflect a complex, context-dependent relationship that cannot be reduced to a simple “beneficial” effect. Therefore, we refrain from drawing mechanistic conclusions from our observational findings.

The lack of association between dietary spermidine intake and prostate cancer incidence, combined with the stage-related finding, is notable. One possible explanation is the homeostatic regulation of polyamines in the human body. Human supplementation studies indicate that after high-dose spermidine supplementation, circulating spermidine levels may not increase significantly, as first-pass metabolism and conversion to spermine, as well as feedback inhibition of endogenous synthesis, buffer the impact of dietary intake (29, 30). This “metabolic buffer” could attenuate any potential effect of diet on tissue polyamine levels, thereby explaining the null incidence finding. For the same reason, the observed stage association- albeit modest and limited to the third quintile-should be interpreted with caution, as it may be influenced by measurement error or residual confounding rather than representing a true biological threshold.

Several methodological considerations warrant caution. First, dietary spermidine intake was assessed using a single baseline food-frequency questionnaire (DHQ), which is subject to non-differential measurement error and does not account for changes in diet over follow-up. This type of error typically biases associations toward the null, potentially underestimating true relationships. Second, spermidine-rich foods (e.g., soy products, whole grains, legumes, mushrooms) are components of healthy dietary patterns that are also rich in fiber, phytochemicals, and other bioactive compounds. Thus, residual confounding by overall diet quality or other lifestyle factors (e.g., physical activity, socioeconomic status) may partly explain the observed associations with all-cause mortality and stage, even after multivariable adjustment (31). Moreover, total energy intake was not included in Model 3 because it could not be reliably calculated from the processed dietary dataset; residual confounding by total caloric intake therefore cannot be excluded. Third, although the PLCO trial randomiZed screening assignment, the dietary analysis remains purely observational; causality cannot be established. Fourth, our exclusion of participants who died from non-prostate cancer causes (as initially described) could introduce selection bias. To address this, we reanalyzed the mortality data using Fine-Gray competing risk models that treat deaths from other causes as competing events; the results remained largely unchanged (no significant association with prostate cancer-specific mortality), but the exclusion approach in the original cohort definition remains a limitation. Fifth, the relatively small number of prostate cancer-specific deaths (n = 603 in the analytic sample) limited statistical power to detect modest hazard ratios; the non-significant elevated HR in Q5 (HR = 1.24, *p* = 0.10) could be due to chance. Sixth, the study population consists predominantly of older White men in the United States, limiting generalisability to other racial/ethnic groups or younger populations. The main strengths of this study include its large sample size, prospective design, long-term follow-up (median follow-up 14.41 years, total person-years 1,778,221), and the ability to examine multiple related endpoints (incidence, stage, all-cause mortality, and cause-specific mortality) within the same cohort. The use of restricted cubic splines allowed for flexible modeling of non-linear dose–response relationships, which may better reflect true nutritional effects than categorical analyses alone (22).

Given the observational design, residual confounding, and measurement error, our findings are hypothesis-generating, not conclusive. The non-linear inverse association between spermidine intake and prostate cancer stage, and the inverse association with all-cause mortality, warrant further research. However, no clinical or dietary recommendations can be made for prostate cancer prevention or management. Any suggestion that spermidine “slows progression” is unsupported, as progression was not measured and the stage association may reflect detection bias or confounding.

Although the sample size (*N* = 54,517; 6,643 cases) provided adequate power to detect HR ≥ 1.15, it may have been underpowered for smaller associations (e.g., HR 1.05–1.10). Thus, null findings for incidence should be interpreted cautiously.

An additional limitation is PSA screening contamination in the PLCO control arm, which may attenuate true incidence associations. Although analyses adjusted for randomization arm (P for interaction = 0.832 for stage; 0.702 for mortality), residual detection bias cannot be excluded.

Future research should prioritize replication of these non-linear associations in independent, ethnically diverse cohorts with different dietary cultures. Whenever possible, studies should incorporate biomarkers of polyamine status (e.g., plasma or tissue spermidine/spermine ratios) to overcome the limitations of dietary self-report and to better understand the relationship between internal exposure and outcomes. Additionally, randomized controlled trials with prostate cancer progression (e.g., biochemical recurrence, metastasis) or quality of life as primary endpoints are needed to test causality. Such trials should consider moderate intake levels (rather than high-dose supplementation) based on the observed non-linear pattern and should include pre-specified analyses for non-linear effects.

## 5 Conclusion

In this large prospective cohort of middle-aged and older men, dietary spermidine intake was not associated with prostate cancer incidence after adjustment for available demographic, lifestyle, and clinical confounders. No independent association was found between spermidine intake and prostate cancer-specific mortality in Fine-Gray competing risk models, although a non-significant trend toward increased risk was observed at the highest intake level. A non-linear inverse association was observed between spermidine intake and all-cause mortality, as well as a non-monotonic association with lower prostate cancer stage limited to moderate intake levels (third quintile). Given the observational design, potential for residual confounding, and lack of association with the primary outcomes of incidence and cancer-specific mortality, these findings should be considered hypothesis-generating and exploratory. They do not support clinical or dietary recommendations for spermidine in prostate cancer prevention or management.

## Data Availability

The raw data supporting the conclusions of this article will be made available by the authors, without undue reservation.
